# A deep learning analysis of stroke onset time prediction and comparison to DWI-FLAIR mismatch

**DOI:** 10.1016/j.nicl.2023.103544

**Published:** 2023-11-16

**Authors:** Ela Marie Z. Akay, Jana Rieger, Ricardo Schöttler, Jonas Behland, Raphael Schymczyk, Ahmed A. Khalil, Ivana Galinovic, Jan Sobesky, Jochen B. Fiebach, Vince I. Madai, Adam Hilbert, Dietmar Frey

**Affiliations:** a Charité Lab for Artificial Intelligence in Medicine (CLAIM), Charité Universitätsmedizin Berlin, Berlin, Germany; b Center for Stroke Research Berlin, Charité Universitätsmedizin Berlin, Berlin, Germany; c Department of Neurology, Max Planck Institute for Human Cognitive and Brain Sciences, Leipzig, Germany; d QUEST Center for Responsible Research, Berlin Institute of Health (BIH), Charité Universitätsmedizin Berlin, Berlin, Germany; e Faculty of Computing, Engineering and the Built Environment, School of Computing and Digital Technology, Birmingham City University, Birmingham, United Kingdom

**Keywords:** Artificial intelligence, Acute ischemic stroke, Machine learning, Decision support, Computer aided, Magnetic resonance imaging, Wake up stroke, DWI-FLAIR-mismatch, Precision medicine, Diffusion-weighted imaging, Fluid attenuated inversion recovery, Cerebrovascular accident, Deep learning

## Abstract

**Introduction:**

When time since stroke onset is unknown, DWI-FLAIR mismatch rating is an established technique for patient stratification. A visible DWI lesion without corresponding parenchymal hyperintensity on FLAIR suggests time since onset of under 4.5 h and thus a potential benefit from intravenous thrombolysis. To improve accuracy and availability of the mismatch concept, deep learning might be able to augment human rating and support decision-making in these cases.

**Methods:**

We used unprocessed DWI and coregistered FLAIR imaging data to train a deep learning model to predict dichotomized time since ischemic stroke onset. We analyzed the performance of Group Convolutional Neural Networks compared to other deep learning methods. Unlabeled imaging data was used for pre-training. Prediction performance of the best deep learning model was compared to the performance of four independent junior and senior raters. Additionally, in cases deemed indeterminable by human raters, model ratings were used to augment human performance. Post-hoc gradient-based explanations were analyzed to gain insights into model predictions.

**Results:**

Our best predictive model performed comparably to human raters. Using model ratings in cases deemed indeterminable by human raters improved rating accuracy and interrater agreement for junior and senior ratings. Post-hoc explainability analyses showed that the model localized stroke lesions to derive predictions.

**Discussion:**

Our analysis shows that deep learning based clinical decision support has the potential to improve the accessibility of the DWI-FLAIR mismatch concept by supporting patient stratification.

## Highlights

• a deep learning model predicts time since stroke onset using unprocessed MRI images.
• the model’s performance is comparable to junior and senior human raters.
• augmenting human decision making with the model improves interrater agreement.
• artificial intelligence can support treatment decisions in acute ischemic stroke.

## 1 Introduction

Acute ischemic stroke is the most common neurological emergency and even though advances in treatment have been made, it remains a leading cause of death and disability worldwide (GBD, 2015, Phipps and Cronin, Feb. 2020). In large prospective randomized trials, the time between symptom onset and treatment using intravenous thrombolysis (IVT) was the main predictor of treatment success. Thus, knowing the time since symptom onset is crucial for deciding which patients to treat with IVT. However, in around 1 in 5 S, the time of onset is unknown, excluding these so-called wake-up stroke patients from receiving IVT treatment (Mackey, May 2011, Moradiya and Janjua, Nov. 2013). The DWI-FLAIR mismatch concept developed by Thomalla et al. is an approach using magnetic resonance imaging (MRI) to stratify these patients for IVT treatment. It states that a lesion visible on diffusion-weighted imaging (DWI) without a corresponding parenchymal hyperintensity on fluid-attenuated inversion recovery (FLAIR) is suggestive of time since stroke <4.5 h and is associated with good IVT treatment outcome (Thomalla et al., 2011). This approach yielded promising results with a balanced accuracy of 0.7 for detection of strokes not older than 4.5 h (Thomalla, 2011) and treating patients according to DWI-FLAIR mismatch led to better outcomes compared to a placebo group in the WAKE-UP trial and follow-up studies (Thomalla et al., 2014, Koga, Dec. 2014). These trials have led to an update of the European Stroke Organisation as well as the American Heart Association guidelines recommending patient stratification for IVT treatment by determining DWI-FLAIR mismatch (Berge et al., 2021, Powers et al., 2019).

While the DWI-FLAIR mismatch concept leads to improvements in patient outcomes (Thomalla et al., 2020), it is limited by immediate availability of MRI imaging and experienced clinicians (Kulzer, Oct. 2021), interrater agreement, and accuracy of the method (Thomalla et al., 2011, Thomalla et al., 2014). Additionally, pre-existing lesions such as white matter hyperintensities or old strokes can further complicate the rating process (Thomalla et al., 2011, Kulzer, Oct. 2021). These difficulties could potentially be remedied by automating the assessment of stroke onset time in the form of a clinical decision support system (CDSS). This would reduce the training required, alleviate the problem of limited interrater agreement, and help achieve maximum accuracy. A suitable method for this type of CDSS would be the application of artificial intelligence (AI). AI methods have been on the rise in various medical fields including neuroradiology and hold great potential for image processing tasks. A machine learning algorithm could serve as a CDSS by performing the DWI-FLAIR mismatch classification task and thereby aiding clinicians in treatment stratification. Deep learning (DL) is a form of machine learning that allows for the detection of higher-level imaging features from raw data using these features to solve a classification task (LeCun et al., 2015). This technique has shown great success in neuroimaging in stroke Soun et al. (2021) and several approaches have shown successful DWI-FLAIR mismatch rating using DL to predict time since onset (Ho et al., 2019, Hyunna et al., 2020, Zhu et al., 2021, Jiang et al., 2022). However, all proposed methods require either additional imaging (Ho et al., 2019) or a significant amount of automated (Hyunna et al., 2020, Zhu et al., 2021) or manual (Jiang, Jan. 2022) post-processing. These requirements limit the clinical feasibility of the proposed methods as they require further time and human expertise (Lees et al., 2010). Furthermore, the proposed methods do not consider the expertise of the clinician in the stroke workflow by simply automating the task of decision making. However, machine learning can also be used to augment human decision making. This allows for an improved performance without removing the clinician’s expertise and factors not provided in the model to make a comprehensive and individualized treatment decision for each patient. In this paper, we propose a DL model using only DWI and FLAIR imaging with minimal post-processing to augment human decision making in by providing stroke onset time classification.

## 2 Methods

### 2.1 Data

#### 2.1.1 Patients

The data used was the 1000plus dataset (Hotter et al., 2009) from a single-center study conducted at the Charité Universitaetsmedizin Berlin on 1472 patients with the clinical diagnosis of acute ischemic stroke and acute DWI and FLAIR images of good quality. For model training and testing, the dataset contained patients with a supratentorial ischemic stroke lesion visible on acute DWI imaging, a documented stroke onset time and acute FLAIR imaging. Another cohort from the 1000plus dataset was created to facilitate pre-training. This included all the patients that had good quality DWI and FLAIR imaging available but did not meet the inclusion criteria, namely, patients with infratentorial stroke lesions, transient ischemic attacks, hemorrhagic stroke, unknown acute event classification, no lesions visible on DWI or unknown stroke onset time.

All experimental protocols of the 1000plus study were approved by the institutional ethical review board of Charité Universitaetsmedizin Berlin. All patients gave written informed consent. All methods were carried out in accordance with the Declaration of Helsinki.

#### 2.1.2 Accessibility

Due to data protection laws, the imaging data used in this study cannot be published at the current time point. Implementation of the proposed network, as well as the training, prediction, and evaluation framework can be found on Github at https://github.com/prediction2020/dwi-flair-mismatch.

#### 2.1.3 Data post-processing

The imaging data was left “raw” without applying any pre-processing steps before coregistration. The FLAIR images were coregistered to the DWI images using SimpleElastix (Lowekamp, Jun. 26, 2015.) and resized to the same size of 192×192×50 voxels. The data was loaded using Nibabel (Brett et al., 2020).

We standardized the voxel intensities to have 0 mean and standard deviation of 1 across the training set. The training set mean and standard deviation were then used to standardize the voxel intensities in the validation and test set.

#### 2.1.4 Qualitative DWI-FLAIR-Mismatch assessment and label generation

Four independent raters blinded to the time of stroke onset visually assessed the presence of DWI-FLAIR mismatch. The raters consisted of two junior raters (EMA, 4 years of experience in stroke imaging and JB, 3 years of experience) and two senior raters (IG, +10 years of experience and AK, 10 years of experience). Raters were instructed to rate the images based on the WAKE-UP study manual (Thomalla et al., 2014) according to the following criteria: a) DWI-FLAIR mismatch was defined by the acute ischemic lesion visible on DWI (DWI-positive) but no marked parenchymal hyperintensity visible on FLAIR (FLAIR-negative) (Thomalla, Aug. 2014), b) In concordance with previous studies into DWI-FLAIR mismatch any FLAIR hyperintensity was rated as FLAIR-positive as long as they were not present in the other hemisphere as to control for extensive leukoaraiosis (Thomalla et al., 2011, Thomalla et al., 2014, Jakubicek, Feb. 2019, Odland et al., Feb. 2015), c) Patients with lesions larger than ⅓ of the MCA, ½ ACA or ½ PCA vessel territory visible on DWI were not included in the analysis (Thomalla et al., 2014, Odland et al., Feb. 2015). d) Contrasting was allowed, but aggressive contrasting to reveal lesions in FLAIR was discouraged (Thomalla et al., 2014). e) Patients were rated as indeterminable if the presence of a FLAIR lesion could not be evaluated e.g., due to extensive leukoaraiosis or other lesions in the area of the stroke lesion.

The label used for supervised deep learning was the time from onset of the stroke until MRI acquisition (time-to-MRI), dichotomized at the commonly used 4.5 h threshold (<= 4.5 h = 1, > 4.5 h = 0). The performance of the deep learning models in predicting the dichotomized time to MRI was compared against the human raters’ performance in DWI-FLAIR mismatch rating.

#### 2.1.5 Deep learning model

**Fig. 1:**
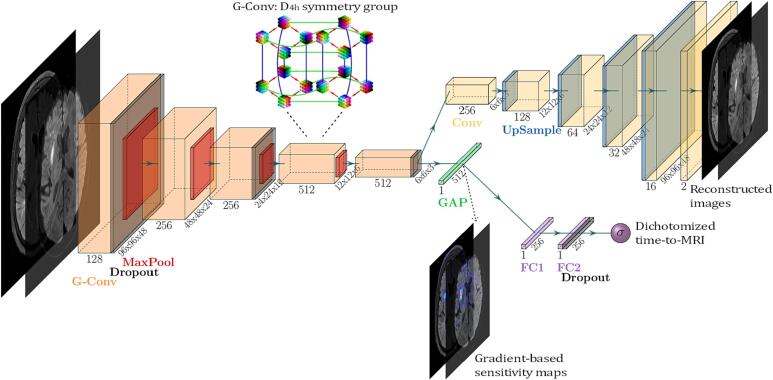
Deep learning architecture, visualized using the PlotNeuralNet software (Iqbal, Dec. 25, 2018.).

For a visual overview of the architecture, see Fig. 1. Our baseline architecture is a Convolutional Neural Network (CNN), consisting of an image encoder and a classifier. The encoder includes five stages, each of them halving spatial dimensions using 3D max-pooling. Global average pooling (GAP) is applied to the output of stage 5 to squeeze the spatial dimensions and create a vector representation. The DWI and FLAIR image input is fed to the encoder together, merged channel-wise, thus creating an input dimension of 192×192×50×2 voxels. The encoder is followed by two fully connected layers with ReLU activation, a dropout and a final classification layer with sigmoid activation.

In the proposed data-efficient variant of this architecture, we use Group Equivariant convolutions (G-convolutions), introduced by Cohen and Welling (Cohen and Welling, 2016). G-convolutions implement equivariance of convolutional kernels towards specified symmetry groups, in our case the $D_{4h}$ symmetry group with 16 orientations and enhance the image feature extraction capabilities of CNNs.

Additionally, we analyzed the impact of exploitation of further imaging data (609 patients) that did not meet the inclusion criteria for time-to-MRI prediction. Unsupervised learning is known to facilitate learning of image features that can be efficiently transferred and utilized as initialization in consecutive supervised tasks improving stability and convergence on limited data. Thus, we constructed a proxy task that enabled the encoder to pre-learn image feature representations in an unsupervised manner. For this, the encoder part of the original architecture was augmented with a bottleneck and a corresponding decoder part, hence creating a Convolutional Autoencoder (CAE) architecture as depicted in Fig. 1. We used the original dataset combined with the additional set of patients, in total 1098 pairs of DWI and FLAIR images. The CAE was trained to optimize the reconstruction of the inputs, and then the pre-trained weights from the encoder were used as initialization for the baseline architecture for time-to-MRI prediction.

Finally, inspired by the work of Myronenko (2018), we exploited the CAE architecture to test the incorporation of reconstruction into the supervised training of time-to-MRI prediction. For this, both prediction and reconstruction outputs were used and aggregated in the training loss, we refer to this method as Autoencoder regularization. We trained two versions of this approach: 1) initializing the architecture with random weights and 2) initializing weights of the encoder through unsupervised pre-training. For technical details concerning the architecture, see the Sup. plementary material.

#### 2.1.6 Model training

A 4-fold cross-validation scheme was applied to increase the robustness of the evaluated models toward different training and evaluation sets. The data was split randomly into distinct training, validation and test sets for each fold with ratios of 56 %, 19 %, and 25 %, respectively, to ensure separation of hyperparameter tuning from model evaluation. Test sets had no overlapping patients. The random splits were determined before all experiments and used equally by all models in each experiment.

For the model training predicting dichotomized time-to-MRI, we employed the Stochastic Gradient Descent (SGD) optimizer with Nesterov momentum (Sutskever et al., 2013) and class-weighted binary cross-entropy as the loss function to deal with class imbalance. The class weights are defined as $w_{0}=\frac{N_{\mathrm{total}}}{2\cdot N_{0}}$; $w_{1}=\frac{N_{\mathrm{total}}}{2\cdot N_{1}}$. Additionally, for the unsupervised pre-training with CAE as well as the AE regularization techniques, Mean Squared Error (MSE) loss function was used. The combined loss function for AE regularization is defined as $L=\alpha L_{\mathrm{bc}}+\left(1-\alpha \right)L_{\mathrm{mse}}$, where α is a loss weighting factor,$L_{\mathrm{bc}}$ is the binary cross-entropy loss function and $L_{\mathrm{mse}}$ is the MSE loss function. For details on hyperparameter tuning, see the sup. plemental material.

A baseline standard CNN was trained as a baseline model.

Computation has been performed on the HPC for Research cluster of the Berlin Institute of Health using an NVIDIA Tesla V100 GPU with 32 GB of VRAM.

#### 2.1.7 Performance assessment

The DL models’ performance was mainly assessed based on the Area Under the Receiver.

Operating Characteristics Curve (AUC). For the DL models, we report mean AUC values and standard deviation across 4 distinct test sets defined by the cross-validation scheme.

Next to AUC, we also report balanced class accuracy (bAcc), sensitivity and specificity. To be able to directly compare performance of human rating and DL predictions, we calculated these metrics for each of the 4, non-overlapping test sets with the corresponding models and report performance on the whole set similarly to human rating. The “indeterminable” rated cases were counted as > 4.5 h since they would generally be excluded from the IVT group. We calculated Cohen’s kappa as a metric for interrater agreement using scikit-learn (Pedregosa, 2011). To calculate bAcc, sensitivity and specificity, the continuous output of DL models were thresholded twice, once using the Youden index (T. S4) in the respective ROC analysis (Ruopp et al., Jun. 2008) and thresholding the DL model’s predictions to the sensitivity of the PRE-FLAIR baseline on the validation set and applying this for test predictions (See Table 2). For performance of human raters augmented with DL predictions: in cases that were rated “indeterminable”, the DL prediction was used; otherwise human rating was counted. We tested statistical significance of the difference 1) between the performance of DL Model and human raters and 2) between the performances of human raters versus ratings augmented with DL prediction by a bootstrapping approach combined with Wilcoxon signed-rank test. We sampled a subset of N patients from the whole dataset, computed each performance metric for human and augmented ratings and repeated this procedure M times with replacement. Statistics were then calculated by the Wilcoxon signed-rank test for each reported metric and statistical significance was determined on a level of p < 0.05. The size of subsets, N, was set to 200 for each rater to ensure a fair representation of indeterminable ratings in the bootstraps, whereas the number of repetitions, M, were set large enough to observe less than 0.01 difference between the average performance metric across bootstraps and the overall performance on the full set.

#### 2.1.8 Explainable artificial intelligence (xAI)

We employed the SmoothGrad (Smilkov et al., 2017) method to produce gradient-based saliency maps, suggesting which areas contributed the most to the given prediction.Human DWI-FLAIR mismatch rating is merely driven by 1) identification of signs of hyperintense lesion on the patient’s DWI and 2) determining the degree of presence of similar traits in the same location on the FLAIR image. Hence, we assessed the rate of attention on DWI lesions as well as the rate of attention in the same location on FLAIR. Optimally, the model should not only recognize hyperintense lesion signs but should derive intuition to test the location of an identified DWI lesion on FLAIR, regardless of high-intensity input from FLAIR imaging.

To assess whether the model focused on the correct hyperintensities in the input images, saliency heatmaps were visually assessed by the junior rater using itkSNAP (Yushkevich et al., 2006) by comparing the original scans and the heatmap side by side. A universal threshold was set by determining the optimal heatmap intensity for the visual rating using three example patient’s images. This threshold was then used for all heatmaps to allow for a comparison between patients. The xAI was rated well-localized if the area with high intensity on the DWI xAI matched most of the hyperintense lesion in the DWI image and the FLAIR xAI covered most of the same location.

## 3 Results

We present a compact version of the performance metrics and results. For details see the sup. plementary material.

### 3.1 Dataset

**Fig. 2:**
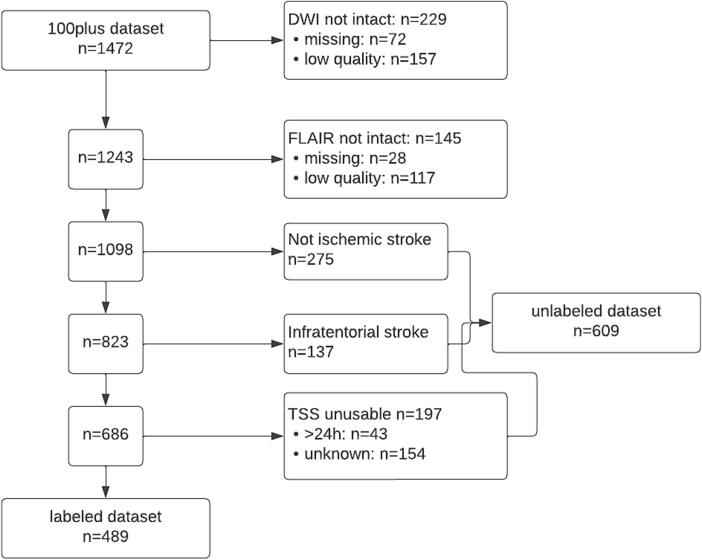
Flowchart of patients used for labeled and unlabeled dataset(DWI = Diffusion-weighted imaging, FLAIR = Fluid-attenuation inversion recovery, TSS = time since stroke).

We included 489 patients with labeled DWI and FLAIR images for the downstream task of time-to-MRI prediction and another 609 patients with unlabeled DWI and FLAIR images for network pretraining on image reconstruction tasks. For a detailed overview of the patient cohorts included and excluded from the dataset, see Fig. 2. For characteristics of the patient cohorts, see Table 1. Demographic information regarding race, ethnicity and socioeconomic status were not recorded in the dataset.

**Table 1:** Demographic overview of patients used in unlabeled, labeled and total dataset (TOAST = Trial of Org 10,172 in Acute Stroke Treatment classification, TIA = transient ischemic attack, ICH = intracranial hemorrhage, SAH = subarachnoid hemorrhage, CVST = cerebral venous sinus thrombosis).

Category		Labeled dataset (n = 489)	Unlabeled dataset (n = 609)	Total (n = 1098)
Age (years)	mean	70.3	66.5	68.07
	std	11.75	14.29	13.64
	min	30	21	21
	max	92	94	94

Sex	Male	298 (60.94 %)	358 (58.78 %)	656 (59.74 %)
	Female	191 (39.06 %)	251 (41.22 %)	442 (40.26 %)

Time since stroke	<= 4.5 h	212 (43.35 %)	78 (12.81 %)	290 (26.41 %)
	> 4.5 h	277 (56.65 %)	306 (50.25 %)	533 (48.54 %)
	Unknown	0	225 (36.95 %)	225 (20.49 %)

Diagnosis	Supratentorial ischemic stroke	489 (100 %)	335 (55.01 %)	825 (75.14 %)
	Infratentorial ischemic stroke	0	147 (24.14 %)	147 (13.39 %)
	TIA	0	141 (23.15 %)	141 (12.84 %)
	ICH	0	6 (0.99 %)	6 (0.55 %)
	SAH	0	1 (0.16 %)	1 (0.09 %)
	CVST	0	1 (0.16 %)	1 (0.09 %)
	Other	0	125 (20.53 %)	125 (11.38 %)
	Unknown	0	1 (0.16 %)	1 (0.09 %)

Thrombolysis	None	362 (74.03 %)	572 (93.92 %)	934 (85.06 %)
	Intravenous	125 (25.56 %)	34 (5.58 %)	159 (14.48 %)
	Intraarterial	2 (0.41 %)	3 (0.49 %)	5 (0.46 %)

TOAST classification of strokes	Arterio-arterial	258 (52.76 %)	179 (29.39 %)	437 (39.8 %)
	Cardioembolic	139 (28.43 %)	94 (15.44 %)	233 (21.22 %)
	Microangiopathic	32 (6.54 %)	45 (7.39 %)	77 (7.01 %)
	Other	4 (0.82 %)	29 (4.76 %)	33 (3.01 %)
	Unknown	14 (2.86 %)	16 (2.63 %)	30 (2.73 %)
	Missing value	42 (8.59 %)	245 (40.23 %)	287 (26.14 %)

**Table 2:** Rater’s performance compared to the PRE-FLAIR baseline, model was calibrated to PRE-FLAIR sensitivity of 0.62 on validation sets. Rater + DL denotes augmentation of indeterminable cases with DL predictions. † denotes statistically significant difference between the performance of the DL Model and any other rating and * denotes statistically significant difference between the performance of human and augmented ratings, both on a significance level of p < 0.05.

Rater	Sensitivity	Specificity	Balanced Accuracy
DL Model	0.608†	0.588†	0.598†
Senior 1	0.439	0.881	0.660
**Senior 1 + DL**	0.514*	0.841*	0.678*
Senior 2	0.415	0.859	0.637
Senior 2 + DL	0.486*	0.838*	0.662*
Junior 1	0.335	0.903	0.619
Junior 1 + DL	0.481*	0.845*	0.663*
Junior 2	0.382	0.859	0.621
Junior 2 + DL	0.434*	0.823*	0.629*
**PRE-FLAIR**	**0.620**	**0.780**	**0.700**

### 3.2 Human rater performance

Human raters achieved a performance more specific but less sensitive than the baseline in the PRE-FLAIR study (Sensitivity = 0.62, Specificity = 0.78) (Thomalla et al., 2011). We can see that Senior 1 outperformed Senior 2 while the junior raters show comparable results. The results for all human raters can be seen in Table 2.

### 3.3 DL model performance

The baseline standard CNN reached an AUC of 0.50 and was outperformed by all the G-CNNs.

**Fig. 3:**
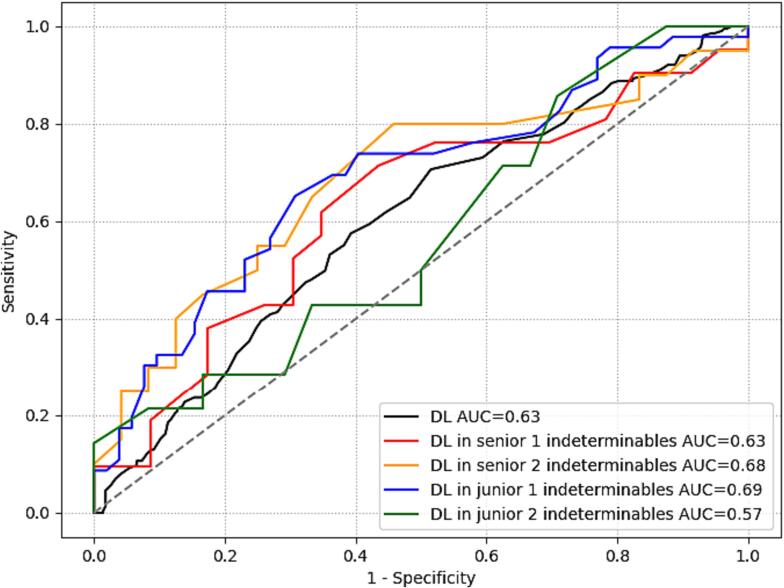
Receiver operating characteristics curve for DL model identifying time since stroke onset within 4.5 h.

Fig. 3 shows the ROC curve for the best DL model (G-CNN with pre-training) with an AUC of 0.63 as well as the model performance on patients deemed indeterminable by the human raters. The model performed comparably well on these patients, with an AUC of 0.69 and 0.57 for the junior raters and AUC of 0.63 and 0.68 for the senior indeterminable patients. In terms of bAcc, the best model performed slightly worse than the human raters (DL 0.60). A comparison of the DL approaches can be seen in T. S3. For a summary of all model performance measures, see T. S4 and S5.

### 3.4 Augmented performance

To augment human performance with DL readings a “second opinion scenario” was simulated using the DL rating in cases where the human rater was unable to determine the mismatch. Since the human raters had significantly higher specificity than the PRE-FLAIR baseline, the main aim of the DL augmentation was to improve on the rating sensitivity. To this end, we calibrated the binarization threshold of the DL model’s predictions to match sensitivity of the PRE-FLAIR baseline on the validation set and applied this threshold for test predictions. Table 2 shows that all the human raters saw an improvement in the balanced Accuracy and sensitivity when augmented by the DL model, while all specificities stayed high above the PRE-FLAIR baseline. The best balanced Accuracy was achieved by augmenting Senior 1 with a balanced Accuracy of 0.68. Augmenting both junior raters led to significant improvements in sensitivity and balanced Accuracy. For Junior 1, DL augmentation surpassed both senior raters in sensitivity. For Junior 2 it resulted in comparable sensitivity to Senior 1 and higher sensitivity than Senior 2. All improvements between the original and augmented human ratings were statistically significant.

### 3.5 Interrater agreement

The interrater agreement between Seniors, Juniors and augmented Juniors can be seen in Table 3. The Senior agreement proved to be good, surpassing the PRE-FLAIR baseline (Thomalla, 2011) and the Junior agreement moderate. When comparing Seniors to augmented Juniors, we can see an improvement in all agreements except for Senior 1 and Junior 2, where agreement did not change when adding DL predictions.

**Table 3:** Interrater agreement as measured by Cohen’s kappa.

Patient groups	Rater 1	Rater 2	Cohen’s ĸ
All patients (n = 489)	Senior 1	Senior 2	0.685
All patients (n = 489)	Junior 1	Junior 2	0.494
Rated by Senior 1 (n = 445)	Senior 1	Junior 1	0.501
Rated by Senior 1 (n = 445)	Senior 1	Junior 1 + DL	0.542
Rated by Senior 1 (n = 445)	Senior 1	Junior 2	0.586
Rated by Senior 1 (n = 445)	Senior 1	Junior 2 + DL	0.586
Rated by Senior 2 (n = 445)	Senior 2	Junior 1	0.464
Rated by Senior 2 (n = 445)	Senior 2	Junior 1 + DL	0.528
Rated by Senior 2 (n = 445)	Senior 2	Junior 2	0.551
Rated by Senior 2 (n = 445)	Senior 2	Junior 2 + DL	0.575
**PRE-FLAIR**			**0.569**

### 3.6 xAI performance and example cases

**Fig. 4:**
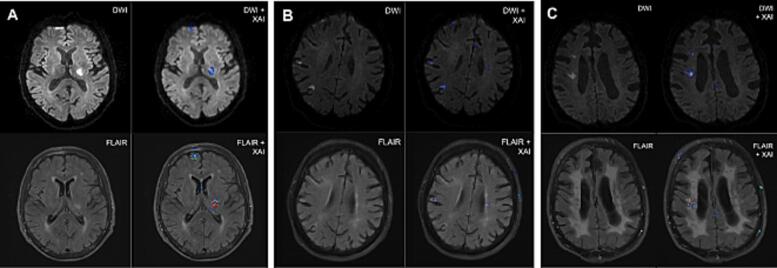
Example xAI image showing a patient with a single infarct (A), scattered infarct (B) pattern and severe leukoaraiosis (C). Top row: DWI image (left), DWI with xAI heatmap overlay (right), bottom row: coregistered FLAIR image (left), with xAI heatmap overlay (right).

The localization performance of the DL approach was tested by the visual rating of xAI heatmaps on the test of the best performing fold with 122 patients. The xAI showed good localization on the lesion in both DWI and FLAIR images in 71.3 % of these cases and in 8.2 % of cases there was good localization on DWI but not on FLAIR. Fig. 4 shows example cases of patients with different infarct patterns and levels of leukoaraiosis which were all classified correctly by the DL model. The regions of interest seem correctly identified but we can see that the infarct was usually highlighted very precisely on DWI images while a larger area around the infarct was highlighted in the FLAIR imaging. We can also see that the xAI shows some highlighted areas corresponding to high-intensity susceptibility artifacts, particularly in the DWI images.

## 4 Discussion

We present a method for acute stroke patient stratification for IVT therapy by using deep learning to predict dichotomized time from symptom onset to MRI (<4.5 h vs > 4.5 h). Our model uses minimal pre-processing and shows promising perspective in augmenting human decisions to improve both interrater agreement and rating performance.

The decision support the model provides is particularly important in cases complicated by additional pathologies on an MRI image that obscure or overlap with the region of interest. These can be localized lesions such as old infarctions and tumors or widespread leukoaraiosis, which are difficult to separate from new ischemic lesions on FLAIR (Thomalla, 2011). While an experienced stroke physician can usually distinguish acute from pre-existing lesions, junior raters might rate these patients as indeterminable, excluding patients from IVT treatment. Our approach provides two advantages in these situations:

Firstly, the model will always provide a rating with comparable accuracy to a human rater as our results show that the model performs particularly well in rating cases deemed indeterminable both by the junior and senior rater. Secondly, it can augment the rating in the indeterminable cases, improving sensitivity and overall performance. Simulating this “second opinion” scenario, we saw that augmenting both junior raters with our DL model predictions improved sensitivity, while preserving specificity above the level of the PRE-FLAIR baseline. The best performance overall resulted from augmenting Senior 1’s rating with the DL predictions, leading to a balanced Accuracy of 0.68.

We can see that interrater agreement measured by Cohen’s kappa improves or remains the same when the cases rated indeterminable by the junior were replaced by the DL prediction and this is compared to the senior rater. In some cases, we achieved agreement above that reported in PRE-FLAIR (ĸ=0.57) (Thomalla, 2011). The augmented agreements outperformed both the interrater agreement described by Galinovic et al. (ĸ=0.47) (Galinovic, Apr. 2014) and Fahed et al. (ĸ=0.43) (Robert, Jan. 2018) suggesting a high quality of agreement overall. This improvement in interrater agreement is of great value for the clinical workflow and improves the validity of the DWI-FLAIR-mismatch approach.

Our model provides a clear recommendation to clinicians and thereby allows for a straightforward diagnostic process. This lower threshold of implementation of the mismatch concept could ultimately lead to more wake-up stroke patients gaining access to treatment. Generally, the percentage of patients receiving IVT treatment is still low at around 10–15 % in Europe and North America (Aguiar de Sousa, Mar. 2019, Otite, Aug. 2021, Willey, May 2013) but more importantly varies significantly amongst patient groups and regions (Demaerschalk, Feb. 2016, Richter, Apr. 2021, Stecksén, Feb. 2012). Wake-up stroke patients are greatly disadvantaged by this, since even considering them for IVT requires readily available machinery and expertise to provide advanced neuroimaging (Berge, Mar. 2021). Recent works have developed methods dependent on additional parameters, e.g. perfusion imaging (Ho et al., Jul. 2019) or manually segmented lesion volumes (Hyunna et al., 2020, Jiang et al., 2022) and while the results are promising, clinical feasibility is limited by the requirement of user input creating new steps within the fast-paced diagnostic workflow. As our approach is only dependent on basic and routinely acquired MRI imaging, it significantly lowers this threshold for clinical applicability. Moreover, we would like to note our approach of calibrating the model’s binarization threshold to the actual clinical goal of improving sensitivity of ratings. Since most DL networks have a continuous output, this step can be freely adapted to the actual clinical team’s needs. We suggest future applications for CDSS consider similar approaches resulting in clinically more applicable and usable solutions.

Another necessity for clinical implementation is users understanding how CDSSs reach their decisions (Markus et al., Jan. 2021). As a first step in this direction, our xAI heatmaps provide post-hoc explanations by visualizing areas of interest the model based its predictions on. In a qualitative analysis of these heatmaps, we saw a high number of heatmaps were well-localized corresponding to hyperintense lesions in DWI and FLAIR images. The heatmaps provide only limited information on how and why an AI uses a specific voxel, however, considering that the model had no input concerning lesion location, this result further validates our approach. In general, we saw better localization in DWI than in FLAIR and locations highlighted on FLAIR covered a larger area surrounding the lesion. This is likely due to the vasogenic edema seen on FLAIR extending beyond the area of cytotoxic edema seen on DWI. xAI heatmaps also reveal factors limiting model performance: In our examples, we see some false-positive voxel attention on DWI corresponding to susceptibility artifacts (e.g., in the frontopolar brain regions, Fig. 4A) with corresponding voxels in the FLAIR image also highlighted. This could lead to a false-positive mismatch and therefore a false prediction of time since onset by the model. In practice, heatmaps can also support the reading of scans and aid the decision-making of junior raters, potentially resulting in improved performances than demonstrated with AI predictions only. Our analysis was exploratory and we encourage prospective validation of DL models following our proposed use case since it has the potential to yield personalized prediction of treatment benefits for wake-up stroke patients. Furthermore, the model is trained only on unprocessed imaging without making immediate assumptions about stroke. This means that the architecture on the model can be easily adapted and used for different brain imaging classification tasks in a transfer learning approach.

Our study has several limitations: First, while the unlabeled dataset used for pre-training originated from the same study, patients had slightly different characteristics (s. Table 1). This could have impeded the extraction of relevant imaging features when the model was initialized with pre-trained weights and might explain the limited performance improvements of the pre-training overall. Second, our image processing pipeline depends on co-registration of the DWI and FLAIR images to allow for comparison of lesion locations amongst images by the model. While fast and readily available implementations exist, this step should be avoided to maximally support the acute setting. Other types of neural networks such as so-called CapsuleNets Sabour et al. (2017) could offer a solution through encoding location information along with extraction of other features. Exploration of these networks on the basis of our work might improve localization and eliminate the need for co-registration. Moreover, it is possible that other deep learning approaches and network architectures might further improve prediction performance. However, extensive exploration of architectures building on this state of the art method was out of the scope of our study. Third, while these results are promising as a proof of concept, the model needs to be externally validated in future projects to ensure robustness of the methods provided. Lastly, even though we achieved promising results in augmenting human decisions, overall prediction performance by the model, it failed to reach the benchmark of the PRE-FLAIR study (Thomalla, 2011). It is important to note however, that while time since onset is a useful proxy to determine IVT treatment success, it remains unclear how dichotomized time since onset corresponds to the potential benefit to patients when treated with IVT as described in the WAKE-UP study (Thomalla et al., 2014). To achieve truly precise and individualized decision support, further validation of our approach is needed, optimally on a more extensive, prospective, multi-center dataset using IVT treatment success.

## 5 Conclusion

We present an artificial intelligence method predicting dichotomized time since stroke onset based on the DWI-FLAIR mismatch concept. This approach shows promise as a clinical decision-aid for IVT treatment stratification by reducing rater uncertainty, increasing interrater agreement and ultimately allowing wake-up stroke patients to gain access to treatment.

**Competing Interests**

A. Hilbert and V. I. Madai reported receiving personal fees from ai4medicine outside the submitted work. D. Frey reported receiving grants from the European Commission and receiving personal fees from and holding an equity interest in ai4medicine outside the submitted work. E. Akay, J. Rieger, R. Schöttler, J. Behland, R. Schymczyk, A. Khalil, I. Galinovic, J. Sobesky and J. B. Fiebach declared no competing interests.

## CRediT authorship contribution statement

**Ela Marie Z. Akay:** Conceptualization, Methodology, Investigation, Writing – original draft, Supervision. **Jana Rieger:** Software, Investigation, Conceptualization, Writing – original draft. **Ricardo Schöttler:** Software, Writing – review & editing. **Jonas Behland:** Investigation, Writing – review & editing. **Raphael Schymczyk:** Software, Writing – review & editing. **Ahmed A. Khalil:** Investigation, Writing – review & editing. **Ivana Galinovic:** Data curation, Investigation, Writing – review & editing. **Jan Sobesky:** Data curation, Writing – review & editing. **Jochen B. Fiebach:** Data curation, Writing – review & editing. **Vince I. Madai:** Conceptualization, Writing – review & editing. **Adam Hilbert:** Conceptualization, Methodology, Investigation, Writing – original draft, Supervision. **Dietmar Frey:** Conceptualization, Project administration, Writing – review & editing.

## Declaration of Competing Interest

The authors declare that they have no known competing financial interests or personal relationships that could have appeared to influence the work reported in this paper.

## Data Availability

The authors do not have permission to share data.
